# Parallel and chain mediating effects of physical activity and aging self-efficacy: community support and subjective well-being among Pre-K–9 teachers in Dongguan

**DOI:** 10.1186/s12889-026-28694-2

**Published:** 2026-07-28

**Authors:** ZhongHan Bao, Dezhuo Sun, Shirong Zhang, Shuqin Chen, Xiangfei Zhu

**Affiliations:** 1 School of Physical Education, Fujian Polytechnic Normal University, Fuqing, Fujian 350300 China; 2 Qishan Campus, MinJiang Teachers College, FuZhou, Fujian 350108 China; 3 Stomatology Campus, Fuzhou First General Hospital, Fuzhou, Fujian 350005 China; 4 Faculty of Liberal Studies, Guangdong University of Science and Technology, Dongguan, 523668 China

**Keywords:** Pre-K–9 teachers, Community Support, Physical Activity, Aging Self-Efficacy, Subjective Well-Being, Dongguan

## Abstract

**Objective:**

To clarify the mediating role of aging self-efficacy and explore how physical activity and community support influence the subjective well-being of Pre-K–9 teachers in Dongguan, a manufacturing city. The aim is to provide empirical evidence in favor of improving the well-being of local teachers.

**Method:**

A questionnaire survey was conducted among 1,005 Pre-K–9 teachers in Dongguan City to collect data, which was then modeled and analyzed using the PLS-SEM model.

**Results:**

Community support, physical activity, aging self-efficacy, and subjective well-being all showed significant positive correlations (*p* < 0.001); community support (β = 0.263, *p* < 0.001) and physical activity (β = 0.289, *p* < 0.001) directly and positively predicted subjective well-being. Furthermore, their positive predictive coefficients for aging self-efficacy were 0.271 and 0.340, respectively (both *p* < 0.001). The independent mediating effect of aging self-efficacy between community support and subjective well-being was 0.053 (*p* < 0.001). The chain mediation effect among community support, physical activity, and subjective well-being was 0.032 (*p* < 0.001). The model explains 47.9% of the variance in subjective well-being.

**Conclusion:**

Subjective well-being of Pre-K–9 teachers in Dongguan can be directly improved by community support and physical activity. Through the twin mediating effects of aging self-efficacy, they also have an indirect promotional effect that is both independent and chained. These findings provide empirical evidence for improving teacher well-being among Pre-K–9 teachers in Dongguan. They may also offer context-specific insights for manufacturing cities with comparable educational structures and teacher demographic characteristics, although further validation in other settings is warranted.

**Supplementary Information:**

The online version contains supplementary material available at https://doi.org/10.1186/s12889-026-28694-2.

## Introduction

### Research background

Subjective well-being is a key indicator for assessing teachers’ physical and mental health, job stability, and long-term commitment to teaching [1]. As a typical manufacturing hub in China, Dongguan has a large migrant population, high population mobility, and complex community structures and governance challenges [2]. The city’s basic education system plays a central social role in ensuring that millions of children of migrant workers receive preschool and compulsory education [3]. Preschool, elementary, and middle school teachers (hereinafter referred to as *Pre-K–9 teachers*) working in this context generally face practical challenges such as teaching in overcrowded classrooms, frequent communication with parents, insufficient community integration, and a relative lack of health-support resources [4]. The physical and mental strain, health-related anxiety, and difficulties in adapting to aging resulting from long-term, high-intensity work are particularly pronounced [5]. Enhancing their subjective well-being has thus become an urgent issue that must be addressed in urban education governance and community development.

In studies on teachers’ subjective well-being, community support, physical activity, and psychological efficacy are all considered key explanatory variables [6]. Community support provides individuals with emotional, practical, and informational resources, serving as a key external factor in alleviating work-related stress and enhancing life satisfaction [7]; physical activity is an effective health-promoting practice for improving physical and mental well-being, alleviating occupational burnout, and fostering positive emotions [8]; Aging self-efficacy refers to individuals’ confidence in maintaining health, preserving functional independence, and adapting successfully to the aging process across midlife and later adulthood, rather than being limited to people who have already entered old age. Previous studies have suggested that concerns about age-related physical decline and future health management often emerge during midlife, particularly among individuals exposed to long-term occupational stress. Therefore, aging self-efficacy has increasingly been applied to middle-aged populations as an indicator of preparedness for healthy aging [9, 10]; it serves as a key psychological mediator linking the external environment to inner well-being. Although there is a close theoretical link between these three factors and subjective well-being, existing research still has significant shortcomings:

Previous research has largely focused on older adults receiving community or healthcare services rather than middle-aged occupational populations [11–13]. Moreover, empirical evidence specifically targeting teachers remains limited.

Firstly, previous studies have typically examined environmental support, health behaviors, or psychological resources separately rather than integrating these factors into a unified environmental–behavioral–psychological framework [14, 15]. This means they fail to adequately elucidate the integrated mechanisms linking environment, behavior, and psychology, which are crucial for understanding how these factors interact in diverse urban settings.

Second, most empirical evidence has been derived from metropolitan areas or regions with relatively abundant educational resources, whereas manufacturing cities remain underrepresented in the literature [16–18].

Third, although physical activity and self-efficacy have each been associated with subjective well-being, few studies have incorporated physical activity and aging self-efficacy into a unified serial mediation framework [12, 19].

Accordingly, this study makes three main contributions to the literature. First, it extends previous research by integrating community support, physical activity, aging self-efficacy, and subjective well-being into a unified environmental–behavioral–psychological framework, rather than examining these factors in isolation. Second, it introduces aging self-efficacy as a theoretically meaningful psychological resource for understanding the well-being of middle-aged teachers, a population that has received limited attention in previous research. Third, by focusing on Dongguan—a manufacturing city characterized by a large migrant population, complex community structures, and substantial educational pressures—this study broadens the contextual evidence base for community health promotion beyond large metropolitan areas and provincial capitals. Therefore, the purpose of this study was to examine how community support influences subjective well-being among Pre-K–9 teachers in Dongguan through the independent and sequential mediating roles of physical activity and aging self-efficacy. Based on the social ecological model and health behavior process theory, a conceptual framework was developed to test the proposed relationships.

### Literature review and research hypotheses

#### Community support and subjective well-being: direct effects

Community support provides individuals with emotional care, practical assistance, information services, and opportunities to participate in activities; it is the most direct external environmental factor influencing an individual’s quality of life and mental health [20, 21]. Numerous studies have shown that consistent and accessible community support can effectively alleviate feelings of loneliness and work-related stress, enhance a sense of security and belonging, and thereby significantly improve subjective well-being [22]. For teachers, community support can also effectively compensate for a lack of support within the school and help mitigate the negative effects of work-related stress [23].

However, existing research has clear limitations: first, it tends to focus on typical cities and areas with strong educational resources while paying little attention to cities dominated by the manufacturing sector, with high concentrations of migrant populations, and where community resources are primarily directed toward basic livelihood support; second, there is still a lack of empirical research targeting teachers from preschool to junior high school that examines the direct impact of community support on subjective well-being. Based on this, we propose the following:

- Hypothesis 1: Community support significantly and directly predicts the subjective well-being of Pre-K–9 teachers in Dongguan in a positive way.

#### The mediating role of physical activity: behavioral pathways

Physical activity is a key health-promoting behavior that improves physical and mental well-being, relieves work-related stress, and boosts positive emotions [24]. Community support can encourage teachers to engage in physical activity by providing fitness facilities, spaces for activities, and an environment that fosters participation [25]; regular physical activity can effectively regulate mood, alleviate fatigue, and enhance life satisfaction, ultimately having a positive impact on subjective well-being [3]. It appears that physical activity may play an important behavioral mediating role between community support and well-being.

However, existing research has two major gaps: First, few studies have specifically examined physical activity as a mediating variable between community support and teacher well-being; second, in manufacturing cities where teachers face high workloads, exercise time is fragmented, and fitness resources are limited, this approach has not yet been supported by empirical evidence. Based on this, we propose:

- Hypothesis 2: Physical activity plays a significant mediating role between community support and subjective well-being.

#### The mediating role of self-efficacy in aging: psychological pathways

Aging self-efficacy refers to an individual’s confidence in their ability to maintain health, adapt to aging, and sustain daily functioning and social participation [26]; it serves as a key psychological mediator linking the external environment to inner well-being [9, 10]. Compared to general self-efficacy, health self-efficacy, or psychological resilience, aging self-efficacy is more closely aligned with the physical and mental characteristics of middle-aged teachers: prolonged, high-intensity work can lead to chronic fatigue, physical discomfort, and health concerns, meaning that an individual’s confidence in maintaining their health and adapting to aging directly influences their life satisfaction and emotional well-being [10].

In typical manufacturing cities, community services tend to focus more on providing basic support for the floating population [3], while health guidance and aging support for teachers are relatively insufficient, making the compensatory role of self-efficacy in aging all the more prominent [27]. Through social engagement, increased emotions of security, and health education, community support can help teachers develop good views about health and aging [28]. High levels of self-efficacy with relation to aging can also improve a person’s sense of control over their life and lessen health-related worry, both of which contribute to subjective well-being [26].

Current research still has significant shortcomings: First, studies on aging adaptation tend to focus on the elderly population, with very little attention paid to middle-aged teachers who are under prolonged stress. Second, no studies have yet examined the mediating role of self-efficacy regarding aging among teachers in manufacturing cities. Based on this, we propose:

- Hypothesis 3: Aging self-efficacy plays a significant mediating role between community support and subjective well-being.

#### The chain mediation effect of physical activity and self-efficacy for aging: an integrated path analysis

According to the social ecological model and the theory of health behavior processes, support from the external environment first influences an individual’s health behaviors, then affects core psychological beliefs, and ultimately impacts mental health [29]. This may contribute to a coherent, comprehensive, and explanatory mechanism: community support → physical activity → self-efficacy regarding aging → subjective well-being.

Community support promotes participation in physical activity by improving access to resources [30]; physical activity enhances self-efficacy regarding aging by improving physical function, enhancing emotional well-being, and increasing a sense of control over one’s health [31]. As a core psychological mediator, self-efficacy regarding aging ultimately enhances subjective well-being. This sequential pathway clarifies how the community environment influences well-being via the combined mediating roles of health behaviors and beliefs about aging, thereby transforming the model from a set of isolated associations into a theoretically coherent integrative framework.

Unlike previous studies that have examined physical activity and aging self-efficacy as separate mediators, the present study proposes that these two mechanisms are hierarchically connected, with physical activity facilitating the development of aging self-efficacy. This sequential conceptualization enables a more comprehensive understanding of the environmental–behavioral–psychological processes linking community support to subjective well-being. Based on this, we propose:

- Hypothesis 4: Physical activity plays a significant chain-mediating role in the pathway from community support to subjective well-being via self-efficacy regarding aging.

Building on the above research gaps, this study investigates the relationships among community support, physical activity, aging self-efficacy, and subjective well-being among Pre-K–9 teachers in Dongguan, China. Specifically, it examines whether physical activity and aging self-efficacy independently mediate the association between community support and subjective well-being and whether these two mechanisms operate sequentially. By integrating environmental, behavioral, and psychological factors into a unified analytical framework, this study extends previous research that has primarily examined these mechanisms separately. The findings are expected to contribute to a more comprehensive understanding of teacher well-being in manufacturing cities and to provide evidence for developing community-based health promotion strategies.

## Research methods

### Research participants

This study limits its scope to teachers in kindergartens, elementary schools, and junior high schools (hereinafter referred to as Pre-K–9 teachers), excluding teachers in secondary vocational schools, primarily based on a comprehensive assessment of Dongguan’s urban functions, educational structure, and research validity:

First, the core functions of urban education differ. Dongguan relies on manufacturing as its pillar industry, and Pre-K–9 teachers play a central social role in ensuring that children of migrant workers receive a basic education; it is a key arena for public services and community governance. Secondary vocational schools focus primarily on developing technical skills; they are smaller in scale and exhibit distinct differences in their social functions and pressure structures.

Second, the professional demands align closely with the research topic. Pre-K–9 teachers commonly face challenges such as large class sizes, frequent communication with parents, long working hours, and a high risk of burnout, which align closely with the core focus of research on community support, physical activity, mental health, and adaptation to aging. Vocational schools are relatively independent and insular, with limited interaction between teachers and the community; therefore, they are not suitable for inclusion in the same model teachers, whose work focuses primarily on practical instruction and skills management, face sources of stress that differ significantly from those of the general population.

Third, there are significant differences in the degree of community cohesion. Pre-K–9 teachers tend to live in industrial parks, urban-rural fringe areas, and newly developed communities and are closely tied to their local environments. Secondary vocational schools are relatively independent and insular, with limited interaction between teachers and the community; therefore, they are not suitable for inclusion in the same model.

Fourth, the sample is more homogeneous, and the study is more targeted. The private sector accounts for a significant share of primary and secondary education in Dongguan, and teachers’ salaries, benefits, and living conditions are more representative of the characteristics of grassroots teachers in a manufacturing city. Excluding secondary vocational teachers ensures consistency in the sample structure, thereby enhancing the reliability of the findings and their applicability to local contexts.

A convenience sampling strategy was employed to recruit Pre-K–9 teachers from kindergartens, primary schools, and junior high schools in Dongguan between September and November 2025. After excluding questionnaires with excessive missing data, patterned responses, or implausibly short completion times, 1,005 valid questionnaires were retained for analysis. Eligible participants were required to (1) be currently employed in a kindergarten, primary school, or junior high school in Dongguan; (2) have worked continuously for at least six months; and (3) voluntarily provide informed consent before participation. Detailed demographic characteristics of the participants are presented in Appendix Table 1 of the Results section.

The eligibility criteria are as follows: (1) Currently employed at a preschool, elementary school, or junior high school in Dongguan; (2) Have worked continuously for 6 months or more; (3) Voluntary participation and informed consent, with a commitment to answer the questions truthfully. Invalid questionnaires—such as those with formulaic responses, excessively short response times, or significant omissions of information—were excluded to ensure data quality.

### Instruments

This study employed adapted structured questionnaires derived from internationally established instruments. To ensure cultural appropriateness for the target population, all scales were translated and culturally adapted according to standardized procedures. First, the original English-language scales were independently translated into Chinese by two bilingual researchers and subsequently back-translated by two additional bilingual experts to ensure conceptual equivalence. Any discrepancies were resolved through discussion until semantic consistency was achieved. Subsequently, five experts in educational psychology, mental health, exercise behavior, psychometrics, and community governance reviewed the translated instruments for linguistic clarity, cultural appropriateness, and content validity. A pilot study involving 85 participants was then conducted to evaluate item clarity, item discrimination, and internal consistency, and minor revisions were made based on item–total correlations and exploratory factor analysis.

Unless otherwise specified, all instruments employed a five-point Likert response format, with higher scores indicating higher levels of the corresponding construct. The psychometric properties of the questionnaire were evaluated in the formal survey. Internal consistency was assessed using Cronbach’s α reliability analysis in SPSS 26.0. Cronbach’s α coefficients ranged from *0.871 to 0.905* (Appendix Table 2), indicating satisfactory reliability. The questionnaire also demonstrated satisfactory validity (0.899; Appendix Table 3), supporting the adequacy of the measurement model.

#### Demographic variables

A demographic information questionnaire, developed in-house, was administered. It covered the following areas: gender; age; household registration type; marital status; educational attainment; years of teaching experience; school type; job nature; average monthly income; and whether the respondent served as a homeroom teacher.

#### Community support

Community support refers to the degree of emotional, instrumental, informational, and participatory support that teachers perceive from community organizations, neighbors, and community public resources (e.g., sports facilities). The scale consisted of four dimensions—Emotional Support, Instrumental Support, Informational Support, and Participatory Support—and 12 items. *The scale was adapted from the Perceived Community Support Questionnaire (PCSQ) developed by Herrero and Gracia (2007)* [32]. *The Cronbach’s α coefficient was 0.902*.

#### Physical activity

Physical activity refers to purposeful, planned, and repetitive physical activities undertaken during leisure time to promote health and improve physical fitness. The scale consisted of four dimensions—Frequency, Intensity, Duration, and Type—and 12 items. *The scale was adapted from Warburton et al. (2006) for PA* [33]. *The Cronbach’s α coefficient was 0.871*.

#### Aging self-efficacy

Aging self-efficacy refers to teachers’ confidence in adapting to age-related changes, maintaining health, preserving independent functioning, and sustaining social participation. The scale consisted of three dimensions—Health Maintenance, Psychological Adaptation, and Social Participation—and 9 items. *The scale was adapted from Suzuki et al. (2023)* [34]. *The Cronbach’s α coefficient was 0.905.*

#### Subjective well-being

Subjective well-being refers to teachers’ overall evaluation of their quality of life, including life satisfaction, positive affect, and negative affect. *The measurement framework was based on Diener’s (1984) conceptualization of subjective well-being* [35]. *The scale comprised three dimensions and 9 items. The Cronbach’s α coefficient was 0.872.*

### Data collection and quality control

Questionnaires were distributed and collected both online and offline from September to November 2025. The research objectives, anonymity principle, and data usage scope were clearly stated at the beginning of each questionnaire to ensure participants’ informed consent. Logical validation checks and time limits were implemented during questionnaire completion. Invalid questionnaires, including those with repetitive responses, randomly filled-out answers within short timeframes, or severe information gaps—were excluded. Valid data underwent double-checking and consolidation by two reviewers to guarantee authenticity and reliability.

### Statistical data analysis

Data processing and analysis were conducted using SPSS 26.0 and SmartPLS 4.1.0.3 statistical software. This included demographic characteristic analysis (Appendix Table 1) and principal component analysis (common method bias test, Appendix Table 4). Correlation analysis was employed to explore relationships among community support, physical activity, aging self-efficacy, and subjective well-being.

Data were analyzed using Partial Least Squares Structural Equation Modeling (PLS-SEM). The analysis proceeded in four stages. First, common method bias was examined. Second, the measurement model was evaluated by assessing indicator reliability, internal consistency, convergent validity, and discriminant validity. Third, the structural model was tested to estimate the hypothesized relationships. Finally, bootstrap procedures were used to examine the direct, indirect, parallel mediation, and serial mediation effects.

In addition, hierarchical regression analysis was performed as a complementary approach to assess the incremental explanatory power of key variables and to verify the robustness of the PLS-SEM results. Specifically, variables were entered in blocks to examine changes in explained variance (ΔR²) in subjective well-being.

## Results

### Measurement model

In accordance with the two-step approach to structural equation modeling, the measurement model was evaluated prior to testing the structural relationships. All measurement properties were examined using SmartPLS version 4.1.0.3. Model estimation was performed using a bootstrapping procedure, and convergence was achieved after seven iterations.

The demographic characteristics of the participants are summarized in Appendix Table 1. Among the 1,005 respondents, 49.5% were male and 49.8% were female. Most participants were aged 46–55 years (64.4%), had more than 21 years of teaching experience (62.0%), and worked full-time (95.5%). Teachers from private kindergartens constituted the largest subgroup (42.7%), and 69.3% reported a monthly income between RMB 3,001 and 8,000.

Table 1 presents the descriptive statistics and Pearson correlation coefficients for the four latent variables: subjective well-being (SWB), physical activity (PA), aging self-efficacy (ASE), and community support (CS). Community support exhibited the highest mean score (M = 3.295, SD = 0.773), while ASE showed the greatest variability (M = 1.248, SD = 0.904). All latent variables were significantly and positively correlated at the *p* < 0.01 level, with correlation coefficients ranging from 0.434 to 0.503, indicating moderate positive associations. These results confirmed the preliminary bivariate relationships and supported the subsequent structural model analysis.

**Table 1:** Correlations among latent variables (*N* = 1005)

Latent variable	Mean	S.D.	SWB	PA	ASE	CS
SWB	3.226	0.812	1			
PA	2.039	0.713	0.503^**^	1		
ASE	1.248	0.904	0.444^**^	0.469^**^	1	
CS	3.295	0.773	0.486^**^	0.479^**^	0.434^**^	1

Std. Deviation (*S.D.*), Aging Self-Efficacy (*ASE*), Community Support (*CS*), Physical Exercise (*PA*), Subjective Well-being (*SWB*)

Table 2 summarizes the results of the measurement model assessment, including standardized factor loadings, Cronbach’s α, composite reliability (CR), and average variance extracted (AVE). All standardized factor loadings ranged from 0.710 to 0.931, exceeding the recommended threshold of 0.7, demonstrating strong item convergence. For internal consistency, Cronbach’s α coefficients for all constructs were between 0.725 and 0.908, and CR values ranged from 0.834 to 0.942, both meeting the 0.7 and 0.8 criteria, respectively. Additionally, the AVE values for all latent variables (0.558–0.845) were greater than 0.5, confirming adequate convergent validity. Collectively, these findings indicated that the measurement model possessed satisfactory reliability and convergent validity.

**Table 2:** Measurement model assessment: indicator loadings, reliability, and convergent validity

Latent variable	Observed variables	Outer loadings	Cronbach’s alpha	CR	AVE
ASE	Health Maintenance	0.904	0.908	0.942	0.845
	Psychological Adaptation	0.931			
	Social Participation	0.922			
CS	Emotional Support	0.811	0.79	0.864	0.613
	Instrumental Support	0.793			
	Information Support	0.781			
	Participation Support	0.747			
PA	Frequency	0.710	0.735	0.834	0.558
	Intensity	0.759			
	Time	0.792			
	Type	0.723			
SWB	Life Satisfaction	0.787	0.725	0.845	0.645
	Positive Emotions	0.818			
	Negative Emotions	0.803			

Aging Self-Efficacy (*ASE*), Community Support (*CS*), Physical Exercise (*PA*), Subjective Well-being (*SWB*), Composite reliability (*CR*), Average variance extracted (*AVE*)

Although the outer loadings for the “frequency” (0.710) and “type” (0.723) indicators of physical activity were relatively lower than those for the “intensity” and “duration” indicators, both exceeded the recommended threshold of 0.70, indicating acceptable indicator reliability. The comparatively lower loadings may reflect the heterogeneous nature of teachers’ physical activity behaviors in Dongguan. Owing to demanding workloads, fragmented leisure time, and differences in access to community exercise resources, teachers may vary considerably in how often they exercise and in the types of activities they choose, whereas exercise intensity and duration tend to be more consistent once physical activity is undertaken. Consequently, greater behavioral variability in exercise frequency and activity type may have reduced their correlations with the latent construct while remaining conceptually important dimensions of physical activity. Consistent with the recommendations of Hair et al., indicators with loadings above 0.70 were retained because they demonstrated adequate indicator reliability, while the construct as a whole exhibited satisfactory composite reliability and convergent validity (CR = 0.834; AVE = 0.558) [36].

Table 3 reports the discriminant validity results assessed via the Fornell–Larcker criterion and the Heterotrait–Monotrait (HTMT) ratio. According to the Fornell–Larcker criterion, the square root of the AVE for each latent variable was consistently larger than its correlation coefficients with all other constructs (e.g., ASE: 0.919 > 0.513 with CS). Furthermore, all HTMT ratios between construct pairs ranged from 0.513 to 0.695, which were well below the conservative threshold of 0.85. These results provided robust evidence of discriminant validity, confirming that the four latent variables were empirically distinct and free from severe multicollinearity issues.

**Table 3:** Discriminant validity assessment using the Fornell–Larcker criterion and HTMT ratios

Fornell-Larcker criterion	ASE	CS	PA	SWB
HTMT				
ASE	**0.919**			
CS	0.513	**0.783**		
PA	0.575	0.633	**0.747**	
SWB	0.548	0.643	0.695	**0.803**

Aging Self-Efficacy (*ASE*), Community Support (*CS*), Physical Exercise (*PA*), Subjective Well-being (*SWB*), Heterotrait-monotrait ratio (*HTMT*)

### Structural model

Table 4 displays the PLS-SEM algorithm settings and overall model fit indices. The algorithm was configured with standard parameters (initial weight = 1, maximum iterations = 3000, stop criterion = 10⁻⁷) and a path weighting scheme, ensuring computational stability and convergence. The model fit indices indicated a good fit between the theoretical model and the data: the standardized root mean square residual (SRMR) was 0.06, which is below the 0.08 cutoff. The d_ULS (0.382) and d_G (0.143) values were within acceptable ranges, and the normed fit index (NFI) was 0.85. The Bayesian information criterion (BIC) values revealed that the model with SWB as the dependent variable (-427.257) had the best fit compared to models with ASE (-310.475) or PA (-254.821) as outcomes.

**Table 4:** PLS-SEM algorithm settings and model fit indices

Category	Indicator	Saturated Model	Estimated Model	Value / Setting
Model Fit	SRMR	0.06	0.06	
	d_ULS	0.382	0.382	
	d_G	0.143	0.143	
	Chi-square	855.354	855.354	
	NFI	0.85	0.85	
Model Selection Criteria (BIC)	ASE			-310.475
	PA			-254.821
	SWB			-427.257
PLS-SEM Algorithm Settings	Initial weights			1
	Maximum number of iterations			3000
	Stop criterion			10⁻⁷
	Type of results			Standardized
	Use Lohmoeller settings			No
	Vary copula by binary categories			Yes
	Weighting scheme			Path

Aging Self-Efficacy (*ASE*), Physical Exercise (*PA*), Subjective Well-being (*SWB*)

**Fig. 1:**
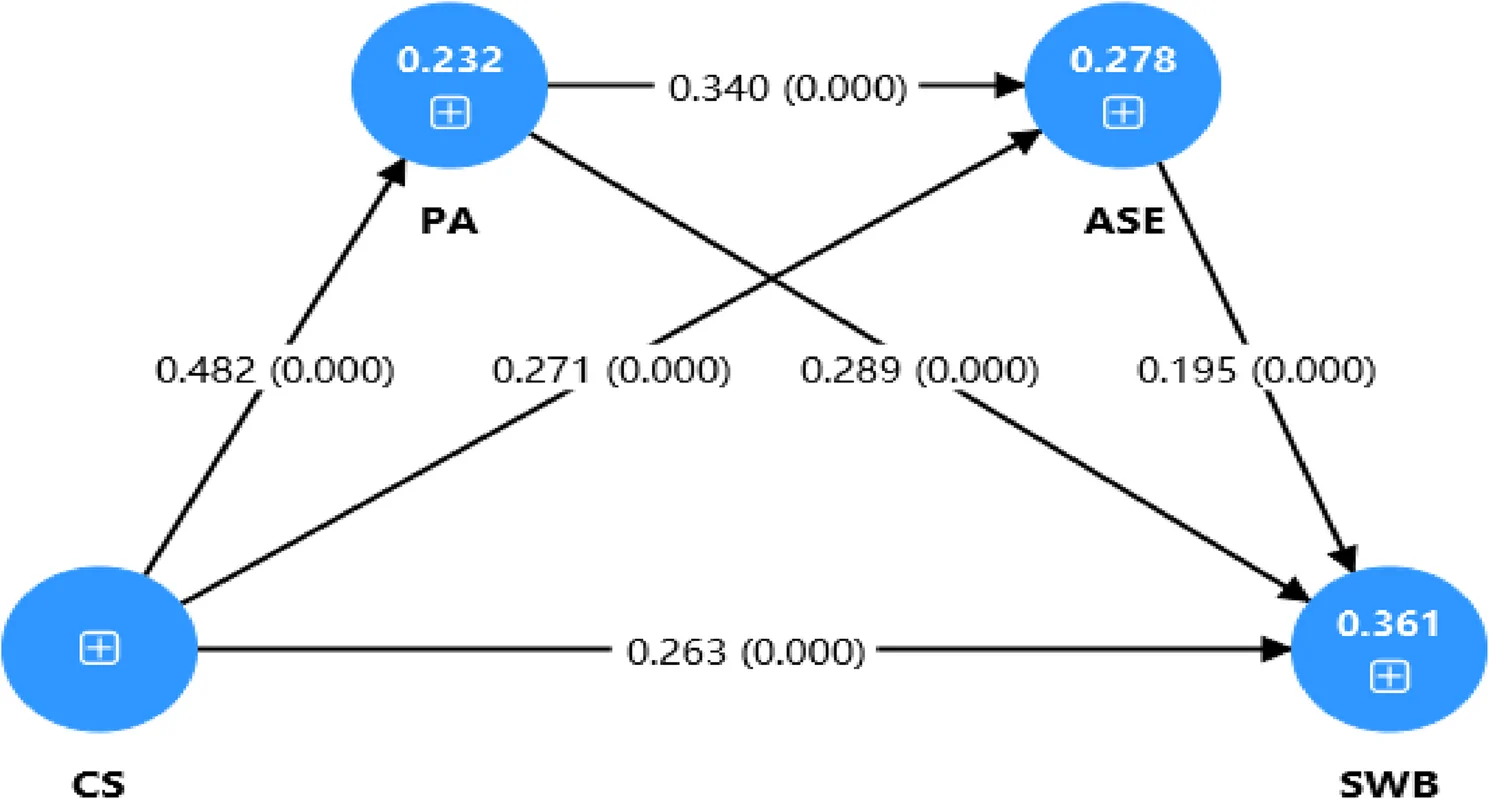
Structural equation model of community support, physical exercise, aging self-efficacy, and subjective well-being among teachers. Note: Aging Self-Efficacy (ASE); Community Support (CS); Physical Exercise (PA); Subjective Well-being (SWB)

Figure 1 presents the results of a structural equation model (SEM) targeting a teacher population, examining the structural relationships among community support (CS), physical activity (PA), aging self-efficacy (ASE), and subjective well-being (SWB). All path coefficients are standardized estimates and reached highly significant levels (*p* < 0.001), indicating strong statistical robustness in the structural relationships among the variables.

First, community support exerts a significant positive influence on physical activity (β = 0.482). In the context of the teaching profession, this finding indicates that teachers are more likely to engage in physical activity when they perceive greater availability of supportive resources from schools, colleagues, and the community. Given the inherent characteristics of the teaching profession—such as high work pressure and fragmented time—support at the community and organizational levels may provide crucial behavioral facilitation environments.

Second, physical activity significantly and positively predicted self-efficacy for aging (β = 0.340). This indicates that among teachers, higher levels of physical activity help enhance individuals’ confidence in their ability to manage their own health and cope with the challenges of occupational aging. For teachers facing career development bottlenecks or accumulated physical and mental burdens, active physical engagement may not only improve physiological conditions but also strengthen their psychological perception of “positive aging.”

Regarding subjective well-being, the model revealed three significant direct paths: community support (β = 0.263), physical activity (β = 0.289), and aging self-efficacy (β = 0.195), all exerting positive effects on subjective well-being. Among these, the direct effect of physical activity was slightly stronger than that of community support, while the effect of aging self-efficacy was relatively smaller yet still significant. This indicates that teachers’ subjective well-being stems both from external support systems and from their own health behaviors and positive beliefs about the aging process.

From a mechanistic perspective, the findings of this study support a chain-mediation model: community support is associated with higher teachers’ subjective well-being by promoting physical activity, which in turn boosts their self-efficacy in aging. This mechanism underscores that teachers’ well-being arises not from a single factor but from the synergistic interplay of social environment, behavioral engagement, and psychological resources.

Table 5 shows the direct and indirect effects of community support (CS) on subjective well-being (SWB), including single and chained mediation effects. Overall, the coefficients for all paths were positive and highly significant (*p* < 0.001), suggesting that the model’s mechanisms are robust.

**Table 5:** Direct, indirect, and chain mediation effects of community support on subjective well-being

Path	β	S.D.	T	*P*	Hypothesis Test
CS -> SWB	0.263***	0.036	7.223	< 0.001	Supported H1
CS -> PA -> SWB	0.139***	0.018	7.862	< 0.001	Supported H2
CS -> ASE -> SWB	0.053***	0.011	4.979	< 0.001	Supported H3
CS -> PA -> ASE -> SWB	0.032***	0.008	4.256	< 0.001	Supported H4

Path coefficients(*β),* Aging Self-Efficacy (*ASE*), Community Support (*CS*), Physical Exercise (*PA*), Subjective Well-being (*SWB*), Standard deviation (*S.D.*), f² values indicate effect sizes following Cohen’s guidelines

First, regarding direct effects, the path coefficient for the CS → SWB path is β = 0.263 (t = 7.223), indicating that community support significantly and positively predicts teachers’ subjective well-being, thereby *confirming H1*. These results indicate that community support retains independent explanatory power even after controlling for mediating variables.

Secondly, with regard to the effect of a single mediator: For the physical activity (PA) mediation pathway (CS → PA → SWB), the effect size was β = 0.139 (t = 7.862). Of all the indirect routes, this had the largest effect size. This *supports hypothesis* *2(H2)*, implying that physical exercise is the most significant mediating factor. The effect size for the mediating path of age-related self-efficacy (ASE) (CS → ASE → SWB) was β = 0.053 (t = 4.979). Although smaller than the PA pathway, it remains significant, *supporting H3* and indicating that psychological and cognitive factors also play a key role.

Furthermore, the route coefficient of the CS → PA → ASE → SWB pathway in the chain mediation effect is β = 0.032 (t = 4.256). This indicates that community support can enhance subjective well-being via a ‘behavior-cognition’ cascade process. This process first may facilitate physical activity and then is associated with increased aging self-efficacy, thereby *supporting H4*.

Table 6*summarizes the explanatory power*,* predictive relevance*,* and effect sizes of the structural model*. The model explained *27.8%* of the variance in aging self-efficacy (ASE), *23.2%* in physical activity (PA), and *36.1%* in subjective well-being (SWB), indicating moderate explanatory power. The adjusted R² values were highly consistent with the corresponding R² values, suggesting good model stability. The Q²predict values ranged from *0.187 to 0.234*, all exceeding zero, thereby confirming satisfactory predictive relevance. In addition, the RMSE and MAE values indicated good out-of-sample predictive performance with relatively small prediction errors.

**Table 6:** Explanatory power, predictive validity, and model fit metrics for the structural model

Latent variable	*R*-square	*R*-square adjusted	Q²predict	f^2^	RMSE	MAE
ASE	0.278	0.277	0.187	CS -> ASE: 0.078; PA -> ASE: 0.123	0.904	0.634
PA	0.232	0.231	0.230	CS -> PA: 0.302	0.88	0.674
SWB	0.361	0.359	0.234	ASE -> SWB: 0.043; CS -> SWB: 0.077; PA -> SWB: 0.089	0.877	0.670

RMSE (root mean square error) and MAE (mean absolute error), Aging Self-Efficacy (*ASE*), Physical Exercise (*PA*), Subjective Well-being (*SWB*)

*The f² effect sizes* further demonstrated the relative contribution of each exogenous construct. According to Cohen’s criteria (0.02 = small, 0.15 = medium, and 0.35 = large), community support exerted a medium effect on physical activity (f² = 0.302), whereas community support (f² = 0.078) and physical activity (f² = 0.123) showed small-to-moderate effects on aging self-efficacy. Likewise, community support (f² = 0.077), physical activity (f² = 0.089), and aging self-efficacy (f² = 0.043) all exerted small but meaningful effects on subjective well-being. Collectively, these findings indicate that the structural relationships are not only statistically significant but also possess meaningful practical effect sizes.

Table 7 presents the results of the hierarchical regression analysis with SWB as the dependent variable, examining the incremental validity of the predictors. Model 1 included only demographic and occupational characteristics, which explained 27.8% of the variance in SWB (F = 42.614, *p* < 0.001). In this model, gender (β = -0.363, *p* < 0.01) was a significant negative predictor, while education (β = 0.439, *p* < 0.001), monthly income (β = 0.536, *p* < 0.001), and holding a leadership position (β = 0.814, *p* < 0.001) were significant positive predictors. Model 2 added CS, resulting in a significant increase in explained variance (ΔR² = 0.127, F change = 211.799, *p* < 0.001), with a total R² of 0.405. CS was a strong positive predictor (β = 0.391, *p* < 0.001). Model 3, the final model, incorporated PA and ASE, leading to a further significant increase in explained variance (ΔR² = 0.074, F change = 70.366, *p* < 0.001) and a total R² of 0.479. Both PA (β = 0.260, *p* < 0.001) and ASE (β = 0.138, *p* < 0.001) were significant positive predictors, and CS remained significant (β = 0.222, *p* < 0.001). All significant predictors across the three models had 95% confidence intervals that did not include zero, confirming the robustness of the effects.

**Table 7:** Multiple regression analysis of factors influencing subjective well-being (SWB)

Model	Observed variables	β	S.E.	T	*P*	LB	UB
1	(Constant)	1.001***	0.240	4.167	< 0.001	0.529	1.472
	Gender	-0.363**	0.107	-3.401	< 0.01	-0.572	-0.153
	Age	-0.122	0.068	-1.795	0.073	-0.255	0.011
	Marital Status	-0.036	0.038	-0.937	0.349	-0.111	0.039
	Educational Background	0.439***	0.063	6.995	< 0.001	0.316	0.563
	Years of Teaching Experience	0.009	0.051	0.180	0.857	-0.091	0.110
	School Type	-0.001	0.027	-0.051	0.959	-0.055	0.052
	Position Type	-0.136	0.081	-1.685	0.092	-0.294	0.022
	Average Monthly Income	0.536***	0.039	13.824	< 0.001	0.459	0.612
	Serves as Homeroom Teacher/Grade Level Coordinator	0.814***	0.095	8.574	< 0.001	0.628	1.000
R Square = 0.278, Adjusted R Square = 0.272							
F = 42.614***, F Change = 42.614***							
2	(Constant)	-0.022	0.229	-0.097	0.923	-0.472	0.427
	Gender	-0.198*	0.097	-2.029	0.043	-0.389	-0.007
	Age	-0.046	0.062	-0.747	0.455	-0.168	0.075
	Marital Status	-0.039	0.035	-1.116	0.265	-0.107	0.029
	Educational Background	0.352***	0.057	6.131	< 0.001	0.239	0.464
	Years of Teaching Experience	-0.015	0.047	-0.322	0.748	-0.106	0.076
	School Type	0.025	0.025	1.003	0.316	-0.024	0.074
	Position Type	-0.077	0.073	-1.049	0.294	-0.221	0.067
	Average Monthly Income	0.426***	0.036	11.831	< 0.001	0.355	0.496
	Serves as Homeroom Teacher/Grade Level Coordinator	0.588***	0.088	6.714	< 0.001	0.416	0.760
	CS	0.391***	0.027	14.553	< 0.001	0.339	0.444
R Square = 0.405, Adjusted R Square = 0.399							
F = 67.658***, F Change = 211.799***							
3	(Constant)	0.111	0.216	0.515	0.607	-0.313	0.535
	Gender	-0.233*	0.091	-2.547	0.011	-0.412	-0.053
	Age	-0.051	0.058	-0.874	0.383	-0.165	0.063
	Marital Status	-0.029	0.033	-0.888	0.375	-0.093	0.035
	Educational Background	0.316***	0.054	5.877	< 0.001	0.211	0.422
	Years of Teaching Experience	-0.018	0.044	-0.414	0.679	-0.104	0.068
	School Type	0.020	0.023	0.843	0.400	-0.026	0.065
	Position Type	-0.056	0.069	-0.809	0.419	-0.190	0.079
	Average Monthly Income	0.367***	0.034	10.759	< 0.001	0.300	0.434
	Serves as Homeroom Teacher/Grade Level Coordinator	0.565***	0.082	6.868	< 0.001	0.403	0.726
	CS	0.222***	0.029	7.651	< 0.001	0.165	0.279
	PA	0.260***	0.032	8.120	< 0.001	0.197	0.323
	ASE	0.138***	0.024	5.669	< 0.001	0.091	0.186

R Square = 0.479, Adjusted R Square = 0.473

F = 75.978***, F Change = 70.366***

a. Dependent Variable: SWB; ***:*P* < 0.001; **:*P* < 0.01; *:*P* < 0.05;

Unstandardized Coefficients (*β*); Std. Error (*S.E.*); 95.0% Confidence Interval for B (Lower Bound, *LB*; Upper Bound, *UB*); Aging Self-Efficacy (ASE); Community Support (CS); Physical Exercise (PA); Subjective Well-being (SWB)

## Discussion

This study, involving 1,005 primary and secondary school teachers in Dongguan, empirically examined the intrinsic relationships among community support, physical activity, aging self-efficacy, and teachers’ subjective well-being using a PLS-SEM model. It validated the independent mediating and chain-mediating effects of aging self-efficacy, drawing upon Dongguan’s unique urban context characterized by its manufacturing-driven economy, dense migrant populations, diverse community structures, and the critical role of primary and secondary education in serving the educational needs of migrant workers’ children. Appendix Table 1 reveals the demographic characteristics of Dongguan’s teaching workforce. 64.4% are aged 46–55, 62.0% have over 21 years’ experience, 42.7% are employed in private kindergartens, and nearly 70% earn below 8,000 yuan per month. These figures closely reflect Dongguan’s foundational education landscape, where private institutions shoulder over half of the educational responsibilities for the children of migrant workers, and experienced teachers form the backbone of basic education, while teacher income levels lag behind the cost of living in Dongguan, a manufacturing city. The findings of this study are not merely theoretical validation but rather an empirical analysis grounded in Dongguan’s urban development realities and tailored to the genuine needs of its teaching community. Its core value lies in providing practical, actionable, and locally relevant solutions to optimize pathways for enhancing teacher well-being and to advance the coordinated development of community services and educational initiatives in Dongguan. Based on the research findings, this discussion focuses on Dongguan’s urban characteristics, teacher needs, and grassroots governance realities. It explores the localized interpretation of core variable relationships, practical pathways for enhancing teacher well-being in Dongguan, and safeguarding mechanisms for grassroots collaboration implementation. The emphasis is on highlighting the practical application value of the research conclusions.

### Localized interpretation of core variable relationships in Dongguan: aligning with urban realities and anchoring teachers’ authentic needs

As a nationally renowned manufacturing hub, Dongguan features over 60% of its economy in processing and manufacturing, hosts a migrant population exceeding ten million, and comprises communities primarily consisting of industrial park rental housing, urban-rural fringe rental districts, and newly built commercial housing complexes [2, 5]. These urban characteristics directly shape the work and life realities of basic education teachers, giving rise to distinctively Dongguan-specific relationships between community support, physical activity, aging self-efficacy, and subjective well-being. The operational mechanisms of all core variables align with Dongguan’s actual development context, rather than representing generalized theoretical conclusions.

#### The “complementary value” of community support: addressing the reality of Dongguan’s high number of private school teachers and weak in-school support

The present study demonstrated that community support significantly and positively predicted both subjective well-being and aging self-efficacy among Pre-K–9 teachers in Dongguan. This finding is consistent with previous studies indicating that perceived community support promotes psychological well-being by providing emotional, informational, and instrumental resources that reduce stress, strengthen social connectedness, and enhance individuals’ coping capacity [32, 37, 38]. These findings also accord with research suggesting that supportive community environments contribute to higher life satisfaction and better mental health among working adults and educators.

However, compared with several previous studies reporting relatively stronger associations between community support and well-being, the direct effect observed in the present study was comparatively modest. This discrepancy may be explained by contextual differences. Many previous studies were conducted in metropolitan areas or communities with relatively mature welfare systems and comprehensive public services, whereas Dongguan is a manufacturing city characterized by a large migrant population and substantial demand for basic public services. Consequently, community resources remain primarily oriented toward housing, employment, and primary healthcare rather than teacher-specific health promotion.

This finding also reflects the characteristics of Dongguan’s educational system. More than half of the teachers in the sample were employed in private schools, with private kindergarten teachers accounting for 42.7%. Many of these schools are located in industrial parks and urban–rural fringe communities with high concentrations of migrant populations. Owing to constrained operating budgets and limited resource allocation, private schools often provide fewer welfare benefits, health promotion programs, and psychological support services than public schools. As a result, teachers rely more heavily on community resources outside the workplace. Nevertheless, community services have not yet fully addressed teachers’ professional health needs. Therefore, the comparatively smaller direct effect of community support should not be interpreted as evidence of limited importance; rather, it suggests that the potential value of community support has not yet been fully realized because existing services remain insufficiently tailored to teachers’ needs.

In addition, nearly 70% of teachers in the present study reported a monthly income below RMB 8,000, whereas housing prices and rental costs in Dongguan remain relatively high. Under these circumstances, market-based services such as psychological counseling, commercial fitness programs, and health management are often financially inaccessible. Community-based universal services, therefore, become an essential rather than optional source of health support. These findings suggest that future community health policies should move beyond meeting the basic needs of migrant populations and place greater emphasis on teacher-oriented health promotion, accessible physical activity opportunities, and psychosocial support services. Such improvements may further strengthen the contribution of community support to teachers’ subjective well-being.

#### The “essential need” attribute of physical activity: aligning with Dongguan teachers’ fast-paced work rhythms and limited fitness resources

The present study found that physical activity exerted the strongest direct effect on aging self-efficacy and a greater direct effect on subjective well-being than community support. *This finding is consistent with previous studies demonstrating that regular physical activity improves psychological well-being by enhancing physical health*,* reducing occupational stress*,* and strengthening self-efficacy* [19, 33, 39]. Previous research has also shown that physical activity contributes to successful aging by improving functional capacity and increasing confidence in maintaining health throughout later life. *However*,* compared with studies conducted among community-dwelling adults or older populations*,* the role of physical activity appeared particularly prominent in the present study*. This difference may reflect the unique occupational characteristics of teachers in Dongguan, where demanding workloads and limited institutional health support make physical activity one of the few accessible strategies for maintaining both physical and psychological well-being.

These findings closely correspond to the working conditions of teachers in Dongguan. Full-time teachers accounted for *95.5%* of the sample, and many reported long working hours, fragmented schedules, and limited opportunities for regular exercise. Teachers are frequently required to manage large classes, communicate extensively with parents, and undertake additional responsibilities such as extracurricular supervision and teaching-related administrative tasks. At the same time, many schools and surrounding communities, particularly those located in industrial parks and urban–rural fringe areas, provide only basic exercise facilities and limited access to organized physical activity programs. Financial constraints may further reduce teachers’ participation in commercial fitness services.

Furthermore, *64.4%* of the participants were aged *46–55 years*, an age group that is more likely to experience work-related fatigue, musculoskeletal discomfort, sleep disturbances, and psychological stress after prolonged teaching careers [40]. Under these circumstances, regular physical activity may serve not only as a means of improving physical fitness but also as an important behavioral resource for strengthening aging self-efficacy and maintaining subjective well-being. This finding extends previous research by suggesting that, among middle-aged teachers, the psychological benefits of physical activity may be particularly pronounced because exercise simultaneously enhances perceived health competence and confidence in adapting to future age-related challenges.

From a public health perspective, these findings suggest that increasing opportunities for regular physical activity may represent one of the most feasible strategies for improving teachers’ well-being in manufacturing cities such as Dongguan. Future interventions should therefore focus on creating accessible community-based exercise programs, improving the availability of school and community sports facilities, and providing professional exercise guidance that accommodates teachers’ demanding work schedules.

#### The “bridging role” of aging self-efficacy: aligning with Dongguan’s reality of a high proportion of mature teachers and prominent health anxiety

Aging self-efficacy significantly and positively predicted teachers’ subjective well-being while also serving as a dual mediator between community support, physical activity, and subjective well-being. *This finding is consistent with previous international research demonstrating that stronger aging self-efficacy is associated with better psychological adjustment*,* healthier behaviors*,* and higher life satisfaction among middle-aged and older adults* [13, 34]. These studies suggest that individuals who are more confident in maintaining their health, adapting to age-related changes, and sustaining social participation are more likely to experience positive psychological outcomes. *However*,* unlike most previous studies*,* which have focused primarily on community-dwelling older adults or retired populations*,* the present study examined aging self-efficacy among actively employed Pre-K–9 teachers*. This difference is theoretically meaningful because the psychological demands of successful aging may emerge well before old age, particularly among teachers who experience prolonged occupational stress, intensive workloads, and increasing concerns about their future health and work capacity.

This finding closely reflects the realities of Dongguan’s aging teaching workforce. In the present study, *64.4%* of teachers were aged *46–55 years*, and *62.0%* had more than *21 years* of teaching experience. This group is at a stage during which age-related physical changes, occupational fatigue, and concerns about future health become increasingly salient. At the same time, teachers in Dongguan often face demanding workloads, uneven access to healthcare resources, and limited community-based health guidance. Under these circumstances, aging self-efficacy—defined as confidence in maintaining health, adapting psychologically, and sustaining social participation—serves as an important psychological mechanism through which external support is transformed into enhanced subjective well-being. In other words, community health resources and participation in physical activity contribute to well-being only when they strengthen teachers’ confidence in aging successfully and coping with future professional and life challenges.

Furthermore, *80.2%* of participants held at least a bachelor’s degree, suggesting relatively high health literacy and receptiveness to health promotion initiatives. This educational background may facilitate the effectiveness of community-based health education and structured physical activity programs in strengthening teachers’ aging self-efficacy. These findings indicate that schools and communities can jointly promote healthy aging by combining accessible physical activity opportunities with targeted health education, thereby reinforcing teachers’ confidence in maintaining health throughout the aging process.

It should also be noted that the concept of aging self-efficacy is not restricted to older adults. Rather, it reflects individuals’ confidence in maintaining health, psychological resilience, and social functioning across the life course. Previous research has shown that perceptions of aging and expectations regarding healthy aging begin to develop during midlife and subsequently influence health behaviors and psychological well-being [12]. Therefore, applying the concept of aging self-efficacy to middle-aged teachers is theoretically appropriate because it captures confidence in adapting to anticipated age-related changes rather than functioning during old age itself. *By extending the application of aging self-efficacy from older populations to middle-aged teachers*,* the present study broadens the theoretical scope of this construct and highlights its value as an important psychological mechanism linking community support and physical activity with subjective well-being in occupational settings*.

#### Practical implications of the chain-mediated pathway: addressing the need for improvement in Dongguan’s school-community collaboration mechanism

The serial mediation pathway identified in this study—community support → physical activity → aging self-efficacy → subjective well-being—extends beyond a theoretical framework by providing practical guidance for strengthening school–community collaboration in Dongguan. At present, educational governance and community governance largely operate independently: schools primarily focus on teaching responsibilities and student management, whereas communities mainly provide general public services for migrant populations. Consequently, teachers’ health promotion receives limited attention from either sector. The present findings suggest that effective collaboration should integrate community-based physical activity resources with school policies that provide teachers with adequate opportunities to participate in regular exercise. Such coordinated efforts may strengthen teachers’ aging self-efficacy and ultimately improve their subjective well-being.

Although the serial mediation effect was statistically significant, its standardized effect size was relatively small. This finding should be interpreted as indicating practical importance. Instead, it reflects the complexity of teachers’ well-being in Dongguan. Unlike the direct and independent mediation effects, the serial pathway requires a sequence of successful transitions, whereby community support first promotes regular physical activity, sustained physical activity enhances aging self-efficacy, and improved aging self-efficacy subsequently contributes to greater subjective well-being. Given teachers’ heavy workloads, fragmented leisure time, and limited access to sustained physical activity, relatively few individuals are likely to complete this entire behavioral–psychological process. Furthermore, limited school–community coordination and insufficient teacher-oriented community health services may further constrain the operation of this sequential mechanism. Therefore, the modest effect size likely reflects the incomplete implementation of supportive environmental conditions rather than the absence of a meaningful behavioral–psychological pathway.

Compared with the independent mediation models, the serial mediation model provides an important theoretical contribution by integrating environmental, behavioral, and psychological mechanisms into a unified explanatory framework. Previous studies have generally regarded physical activity and aging self-efficacy as parallel mediators. In contrast, the present findings demonstrate that physical activity also functions as an antecedent of aging self-efficacy, suggesting that regular participation in physical activity gradually strengthens teachers’ confidence in maintaining health, adapting to age-related changes, and sustaining social participation. This hierarchical relationship provides a more comprehensive explanation of how community support ultimately promotes subjective well-being among middle-aged teachers.

From a public health perspective, these findings suggest that interventions should not address physical activity and aging self-efficacy separately. Instead, community-based health promotion programs should combine supportive environments with sustainable physical activity opportunities, thereby simultaneously enhancing behavioral engagement and psychological resources. Such integrated interventions may generate greater and more sustainable improvements in teachers’ subjective well-being than strategies targeting either mechanism alone.

### Practical pathways for enhancing teacher well-being in Dongguan

Based on the empirical findings, enhancing teacher well-being in Dongguan should prioritize *community-based support*,* school-level facilitation*,* and government coordination*, focusing on feasible, low-cost, and resource-efficient interventions. Three core pathways are proposed: improving targeted community support, optimizing school participation conditions, and strengthening policy-level coordination.

#### Community level: tailor support resources to make communities “life service stations” for teachers

Communities should function as accessible support nodes for teachers by leveraging existing facilities and minimizing additional costs. First, community sports and health resources should be integrated according to local contexts (industrial park communities, rental communities, and commercial housing areas), with simple fitness equipment, shared facilities, and time-based activity groups (e.g., walking or stretching sessions) to promote regular participation.

Second, community service centers should provide basic support services for teachers, including health consultation, stress management information, and community activity updates, thereby improving informational and emotional support.

Thirdly, a low-cost, routine health education system for the elderly should be introduced through existing community platforms. This could include monthly health lectures, quarterly free clinics, and ongoing digital health messages. The aim of this is to improve teachers’ health awareness, support early health screening, reduce health-related stress, and enhance aging self-efficacy.

#### School level: collaborating with communities to make schools “work support stations” for teachers

Schools should focus on removing structural constraints that limit teacher participation in physical and community activities. First, schools should allocate short daily time slots (15–20 min) for group physical activities to address time scarcity. Flexible scheduling arrangements can be provided for high-workload teachers (e.g., homeroom teachers) to support weekly participation in health-related activities.

Second, school sports facilities should be opened to teachers beyond teaching hours, and in resource-limited schools, partnerships with nearby communities can be established to ensure accessible exercise spaces, with minimal financial subsidies when necessary.

Third, schools should establish liaison mechanisms with communities to regularly share activity resources and jointly organize school–community health or sports events, improving participation channels and engagement continuity.

#### Government level: strengthen top-level guidance and resource support to position the government as the “binder for school-community collaboration”

The government should play a coordinating and guiding role by establishing clear institutional arrangements and ensuring equitable resource distribution. First, policy documents should clearly define the division of responsibilities between schools and communities and incorporate teacher well-being indicators into evaluation systems to ensure implementation accountability.

Second, targeted funding mechanisms should be established to support teachers in private schools and under-resourced areas, particularly in health checkups, transportation subsidies, and community sports infrastructure development. In addition, digital platforms should be developed to integrate information on community services and school-based resources, improving accessibility and service efficiency.

Third, differentiated guidance should be applied across urban, industrial, and developing districts to ensure context-specific implementation, avoiding uniform or inefficient investment patterns.

#### Dongguan’s support mechanism for school-community collaboration implementation: addressing grassroots execution challenges to ensure effective measures

Dongguan’s grassroots governance faces practical challenges, including insufficient community staff, heavy teaching workloads in schools, and high communication costs between schools and communities. For teacher welfare enhancement initiatives to be effectively implemented, a simple, efficient, and grassroots-oriented support mechanism must be established. This addresses the issue of “good policies but difficult execution,” preventing measures from becoming mere formalities.

#### Streamline workflows to reduce frontline implementation costs

Both community-school and school-community coordination efforts adopt “checklist-based management.” The Dongguan Education Bureau and Civil Affairs Bureau jointly developed the School-Community Collaboration Checklist, specifying monthly tasks (e.g., one coordination meeting, one sports activity, one health information push). This eliminates the need for frontline units to develop additional plans or prepare materials—they simply follow the checklist to reduce administrative burdens.

#### Integrating grassroots personnel to strengthen school-community collaboration

Create “Teacher Welfare Service Teams” by utilizing pre-existing community grid workers, volunteers, and Party-Mass Service Center employees in Dongguan, in addition to school union representatives, school doctors, and physical education instructors. In addition to facilitating collaboration between the school and the community, these teams will plan physical activities and distribute health information. This approach eliminates the need for additional staff or positions by achieving “dedicated personnel for specific roles, each fulfilling their respective responsibilities.”

#### Establish a regular supervision mechanism to ensure implementation of measures

A special supervision team jointly established by the Dongguan Education Bureau and the Civil Affairs Bureau will conduct quarterly spot checks on school-community collaboration efforts across townships (industrial parks). Key inspection areas include the following: whether teachers’ physical activity time is being implemented, whether community teacher service windows are functioning normally, and whether school-community coordination has been normalized. Inspection results will be circulated citywide, with townships (industrial parks) failing to implement measures effectively subject to formal discussions and rectification. Concurrently, a teacher satisfaction evaluation mechanism will be established. Teachers will be organized to evaluate community and school welfare services every six months, with evaluation results serving as a key basis for performance assessments.

#### Establish exemplary models to drive citywide implementation through spot-to-area expansion

Three representative towns/subdistricts—Humen Town (manufacturing hub), Nancheng District (main urban area), and Liaobu Town (urban-rural fringe)—were selected to pilot school-community collaboration initiatives for enhancing teacher welfare. By summarizing pilot experiences and developing replicable, scalable demonstration models, these approaches will be rolled out across all towns/subdistricts (and industrial parks) citywide. This phased implementation avoids a one-size-fits-all approach, ensuring measures align with each locality’s specific context.

### Research limitations and future directions

#### Research limitations

First, this study employed convenience sampling, and the sample was not proportionally stratified across different regions of Dongguan. In addition, teachers from private kindergartens constituted *42.7%* of the participants, resulting in a sample that may not fully represent the broader teaching population. Because teachers working in different educational settings may differ in employment stability, organizational support, work-related stress, income, and reliance on community resources, combining these groups into a single analytical model may have obscured potential heterogeneity in the proposed mediation pathways. Therefore, caution should be exercised when generalizing the findings to teachers from other educational contexts or geographic regions. Future studies should employ stratified sampling and multi-group structural equation modeling to examine whether the proposed relationships differ across school types and employment contexts.

Second, the cross-sectional design precludes definitive causal inference. Although the hypothesized mediation model was developed based on established theoretical frameworks and previous empirical evidence, reverse or reciprocal relationships cannot be excluded. For example, teachers with higher subjective well-being or stronger aging self-efficacy may be more likely to engage in physical activity, perceive greater community support, and actively utilize community resources. Consequently, the observed associations should be interpreted as theory-consistent rather than causal. Longitudinal and intervention studies are warranted to establish temporal ordering and causal mechanisms.

Third, although the proposed model incorporated key environmental, behavioral, and psychological variables, other potentially important determinants of teachers’ subjective well-being—including occupational stress, burnout, sleep quality, chronic health conditions, organizational support, and personality characteristics—were not included. The omission of these variables may have introduced residual confounding and limited the explanatory power of the model. Future studies should incorporate a broader range of individual, occupational, and organizational factors to further refine the proposed framework.

Finally, because the sample consisted predominantly of middle-aged teachers, aging self-efficacy in the present study should be interpreted as confidence in maintaining health and adapting successfully to future age-related changes rather than adaptation during old age itself. Further research comparing middle-aged and older teachers would help determine whether the proposed structural relationships vary across different stages of the aging process.

#### Future directions

To address the limitations of this study, future research should focus on three areas. Firstly, a stratified random sample should be selected based on Dongguan’s urban districts, townships, industrial parks, and school types (public/private and kindergarten/primary/secondary) to ensure a balanced sample structure.

Future studies should compare mediation mechanisms across teachers from public and private schools, different educational levels, and different employment systems to determine whether organizational context moderates the relationships among community support, physical activity, aging self-efficacy, and subjective well-being. Such analyses would help determine whether the proposed environmental–behavioral–psychological pathway is invariant across educational settings or whether different organizational environments require context-specific intervention strategies.

Second, conduct cross-city comparative studies in similar manufacturing cities, such as Foshan and Suzhou, to validate the applicability of the research model. Secondly, a longitudinal tracking design was employed to conduct a 3–5-year follow-up study of mature teachers aged 46–55 in Dongguan. By collecting panel data, we revealed the dynamic causal relationships among the core variables, analyzing the long-term effects of community support and physical activity on their perceived ability to cope with aging and their subjective well-being. Third, optimize the measurement of variables by combining subjective scales with objective data to assess physical activity. For example, integrate fitness app data and community sports facility usage records to enhance measurement accuracy. At the same time, incorporate qualitative research methods by conducting in-depth interviews with teachers who have a variety of demographic characteristics in order to enrich the depth and breadth of the study.

## Conclusion

Based on the current structure of Dongguan’s manufacturing-dominated economy, where shift workers are predominantly middle-aged and young adults and their children are concentrated in kindergartens, elementary schools, and junior high schools, this study focuses its analysis on Pre-K–9 teachers, who face the most significant stress during this stage.

This study demonstrates that community support is associated with higher the subjective well-being of Pre-K–9 teachers in Dongguan through both direct pathways and the parallel and sequential mediating roles of physical activity and aging self-efficacy. These findings underscore the importance of integrating community resources with health-promoting behaviors and psychological resources to support teacher well-being. However, because the sample consisted predominantly of middle-aged teachers and teachers employed in private kindergartens, the findings should be interpreted within this specific demographic and educational context. Although the proposed framework may provide useful insights for manufacturing cities with similar educational systems and teacher profiles, further studies involving more diverse teacher populations and regional settings are required before broader generalization can be made.

## Supplementary Information

Supplementary Material 1.

Supplementary Material 2.

Supplementary Material 3.

Supplementary Material 4.

Supplementary Material 5.

Supplementary Material 6.

## Data Availability

The datasets generated and/or analyzed during the current study are not publicly available due to privacy and confidentiality concerns. However, they are available from the corresponding author upon reasonable request.
