# Supplementary material for: Parallel and chain mediating effects of physical activity and aging self-efficacy: community support and subjective well-being among Pre-K–9 teachers in Dongguan
Source: BMC Public Health. 2026 Jul 28;26:3312. doi: 10.1186/s12889-026-28694-2 (PMC13753292; doi:10.1186/s12889-026-28694-2)
Supplement: Supplementary file 1 [file 12889_2026_28694_MOESM6_ESM.pdf]

Ethics Approval

Project Title: Aging Self-Efficacy Mediates the Links Between Community Support, Physical Activity, and Teachers' Subjective Well-Being in Dongguan

Registration Number: 2025A146

Date of Registration: March 26, 2025

Ethical Review and Approval

This study was reviewed and approved by the Ethics Committee of Guangdong University of Science and Technology (Ethics Approval Number: 2025A146). The study adheres to ethical standards and guidelines set by the Declaration of Helsinki and local institutional requirements.

Research Participants

The study targeted active teachers in kindergartens, elementary schools, junior high schools, senior high schools, and secondary vocational schools within Dongguan City. A **convenience sampling method** was used to recruit participants, and the following inclusion criteria were applied:

- **Inclusion Criteria:** Participants must be currently employed as teachers in the selected educational institutions and have worked continuously in Dongguan City for at least six months.
- **Exclusion Criteria:** University faculty were excluded due to the distinct industrial structure, demographic characteristics, and educational ecosystem in Dongguan, which may differ significantly from that of primary and secondary education sectors.

Informed Consent

Informed consent was obtained from all participants prior to their involvement in the study. The informed consent process included:

- Detailed information about the research objectives, methodology, and the voluntary nature of participation.
- An assurance of participant anonymity and confidentiality.
- Clarification of the participants' right to withdraw from the study at any time without any penalty.

Participants were asked to sign written informed consent forms, acknowledging their understanding of the study's aims and procedures. All participants were also informed about the measures taken to protect their privacy and the confidentiality of the data collected.

Data Collection and Ethical Considerations

Data collection took place between September and November 2025. Questionnaires were distributed both **online** and **offline** to accommodate the participants' availability and preferences. All participants were informed about the data usage and confidentiality prior to completing the questionnaires.

- **Confidentiality:** Personal information and responses were kept confidential and were not shared with any third parties. Only aggregated data were used for analysis and reporting.
- **Anonymity:** No identifiable information was collected on the questionnaires, ensuring participant anonymity.

Quality Control Measures

To ensure data validity and reliability:

- **Logical Validation Checks:** Automated and manual validation checks were implemented during questionnaire completion to detect inconsistencies or outliers.
- **Quality Control during Data Entry:** Invalid questionnaires (e.g., those with random or incomplete answers) were excluded from analysis. Additionally, surveys completed in an abnormally short time span or with apparent patterns of carelessness were also excluded.
- **Double-checking:** All valid data were independently reviewed by two researchers for verification and consolidation, ensuring the integrity of the data used in the study.

Risk and Benefit Assessment

The study posed minimal risk to participants, focusing primarily on subjective well-being and aging self-efficacy, with no invasive procedures involved. The benefits of the study include providing valuable insights into factors that promote teachers' well-being and health as they age, contributing to the development of future community health interventions.

Seal of Approval: Guangdong University of Science and Technology

Date of Approval: May 6, 2025
