# Supplementary material for: Parallel and chain mediating effects of physical activity and aging self-efficacy: community support and subjective well-being among Pre-K–9 teachers in Dongguan
Source: BMC Public Health. 2026 Jul 28;26:3312. doi: 10.1186/s12889-026-28694-2 (PMC13753292; doi:10.1186/s12889-026-28694-2)
Supplement: Supplementary file 2 [file 12889_2026_28694_MOESM1_ESM.docx]

**Appendix Table 1**. Demographic Characteristics of the Sample Population (N=1005)

| **Variable** | **Category** | **Frequency** | **Percent** |
| --- | --- | --- | --- |
| Gender | Male | 501 | 49.5 |
|  | Female | 504 | 49.8 |
| Age | 22–35 years | 16 | 1.6 |
|  | 36–45 years | 324 | 32.0 |
|  | 46–55 years | 652 | 64.4 |
|  | >56 years | 13 | 1.3 |
| Marital Status | Unmarried | 251 | 24.8 |
|  | Married | 690 | 68.1 |
|  | Divorced | 48 | 4.7 |
|  | Widowed | 16 | 1.6 |
| Educational Background | Associate Degree or Below | 192 | 19.0 |
|  | Bachelor's Degree | 747 | 73.7 |
|  | Master's Degree and Above | 66 | 6.5 |
| Years of Teaching Experience | 5 years or less | 31 | 3.1 |
|  | 6–10 years | 35 | 3.5 |
|  | 11–20 years | 311 | 30.7 |
|  | 21 years or more | 628 | 62.0 |
| School Type | Public kindergartens | 36 | 3.6 |
|  | Private kindergartens | 433 | 42.7 |
|  | Public elementary schools | 237 | 23.4 |
|  | Private elementary schools | 155 | 15.3 |
|  | Public middle schools | 131 | 12.9 |
|  | Private middle schools | 13 | 1.3 |
| Position Type | Full-time teachers | 967 | 95.5 |
|  | Administrative and teaching assistants | 23 | 2.3 |
|  | Support staff and healthcare | 15 | 1.5 |
| Average Monthly Income | ¥3,001–5,000 | 269 | 26.6 |
|  | ¥5,001–8,000 | 433 | 42.7 |
|  | ¥8,001–12,000 | 294 | 29.0 |
|  | ¥12,001 and above | 9 | 0.9 |
| Serves as Homeroom Teacher/Grade Level Coordinator | Yes | 335 | 33.1 |
|  | No | 670 | 66.1 |

**Appendix Table 1** provides a detailed breakdown of the demographic characteristics of the sample population (N=1005), categorizing key variables such as gender, age, marital status, educational background, teaching experience, school type, position type, monthly income, and whether individuals serve as homeroom teachers or grade-level coordinators.

The sample is almost evenly split between male (49.5%) and female (49.8%) participants, with a slight majority of females. In terms of age, the distribution is heavily concentrated in the 46–55 years category, with 64.4% of participants falling within this range. Only a small proportion of participants are in the younger (22–35 years) and older (over 56 years) categories, representing 1.6% and 1.3%, respectively.

Regarding marital status, the majority of respondents are married (68.1%), followed by unmarried individuals (24.8%). A smaller proportion is divorced (4.7%) or widowed (1.6%). As for educational background, most participants hold a bachelor's degree (73.7%), while fewer have an associate degree (19.0%) or a master's degree and above (6.5%).

In terms of teaching experience, the majority have 21 years or more (62.0%), with 30.7% having 11–20 years. Only a small percentage has 5 years or less (3.1%) or 6–10 years of experience (3.5%). The sample is predominantly composed of teachers working in private kindergartens (42.7%) and public elementary schools (23.4%). Smaller proportions work in other types of schools, with private middle schools representing just 1.3% of the sample.

The overwhelming majority of respondents are full-time teachers (95.5%), while only a small proportion holds administrative and support roles (2.3% and 1.5%, respectively). In terms of income, most participants earn between ¥5,001 and ¥8,000 per month (42.7%), followed by those earning ¥8,001 to ¥12,000 (29.0%). Only a small fraction earns ¥12,001 or above (0.9%).

Finally, about one-third of participants (33.1%) serve as homeroom teachers or grade-level coordinators, while the majority (66.1%) do not.
