# Supplementary material for: Parallel and chain mediating effects of physical activity and aging self-efficacy: community support and subjective well-being among Pre-K–9 teachers in Dongguan
Source: BMC Public Health. 2026 Jul 28;26:3312. doi: 10.1186/s12889-026-28694-2 (PMC13753292; doi:10.1186/s12889-026-28694-2)
Supplement: Supplementary file 3 [file 12889_2026_28694_MOESM2_ESM.docx]

**Appendix Table 2**. Internal Consistency Reliability Analysis of Measurement Scales (Cronbach’s Alpha)

| **Variable** | | **Item** | **Rotated Component** | **CITC** | **CAID** | **Cronbach’s α** | |
| --- | --- | --- | --- | --- | --- | --- | --- |
| SWB | Life Satisfaction | LS 1 | 0.727 | 0.745 | 0.811 | 0.865 | 0.872 |
|  |  | LS 2 |  | 0.685 | 0.862 |  |  |
|  |  | LS 3 |  | 0.808 | 0.749 |  |  |
|  | Positive Emotions | PE 1 | 0.780 | 0.690 | 0.738 | 0.821 |  |
|  |  | PE 2 |  | 0.674 | 0.754 |  |  |
|  |  | PE 3 |  | 0.661 | 0.767 |  |  |
|  | Negative Emotions | NE 1 | 0.710 | 0.758 | 0.777 | 0.858 |  |
|  |  | NE 2 |  | 0.708 | 0.824 |  |  |
|  |  | NE 3 |  | 0.732 | 0.802 |  |  |
| PA | Frequency | F 1 | 0.657 | 0.827 | 0.906 | 0.911 | 0.871 |
|  |  | F 2 |  | 0.856 | 0.855 |  |  |
|  |  | F 3 |  | 0.847 | 0.863 |  |  |
|  | Intensity | I.1 | 0.701 | 0.717 | 0.733 | 0.810 |  |
|  |  | I.2 |  | 0.717 | 0.718 |  |  |
|  |  | I.3 |  | 0.723 | 0.792 |  |  |
|  | Time | Time 1 | 0.731 | 0.568 | 0.766 | 0.784 |  |
|  |  | Time 2 |  | 0.647 | 0.682 |  |  |
|  |  | Time 3 |  | 0.658 | 0.672 |  |  |
|  | Type | Type 1 | 0.682 | 0.598 | 0.792 | 0.803 |  |
|  |  | Type 2 |  | 0.660 | 0.724 |  |  |
|  |  | Type 3 |  | 0.720 | 0.651 |  |  |
| ASE | Health Maintenance | HM | 0.867 | 0.788 | 0.884 | 0.905 | |
|  | Psychological Adaptation | PA | 0.868 | 0.833 | 0.846 |  | |
|  | Social Participation | SP | 0.854 | 0.813 | 0.863 |  | |
| CS | Emotional Support | ES 1 | 0.758 | 0.769 | 0.835 | 0.882 | 0.902 |
|  |  | ES 2 |  | 0.760 | 0.843 |  |  |
|  |  | ES 3 |  | 0.784 | 0.821 |  |  |
|  | Tool Support | TS 1 | 0.760 | 0.786 | 0.772 | 0.865 |  |
|  |  | TS 2 |  | 0.745 | 0.809 |  |  |
|  |  | TS 3 |  | 0.701 | 0.849 |  |  |
|  | Information Support | IS 1 | 0.715 | 0.671 | 0.833 | 0.849 |  |
|  |  | IS 2 |  | 0.750 | 0.757 |  |  |
|  |  | IS 3 |  | 0.732 | 0.775 |  |  |
|  | Participation Support | PS 1 | 0.703 | 0.779 | 0.793 | 0.870 |  |
|  |  | PS 2 |  | 0.696 | 0.868 |  |  |
|  |  | PS 3 |  | 0.783 | 0.788 |  |  |

Note: Corrected Item-Total Correlation (CITC); Cronbach's Alpha if Item Deleted (CAID); Aging Self-Efficacy (ASE); Community Support (CS); Physical Exercise (PA); Subjective Well-being (SWB);

**Appendix Table 2** provides an internal consistency reliability analysis for the measurement scales, including Cronbach’s Alpha (α), Corrected Item-Total Correlation (CITC), and Cronbach’s Alpha if Item Deleted (CAID). The analysis covers four key constructs: **Subjective Well-Being (SWB)**, **Physical Activity (PA)**, **Aging Self-Efficacy (ASE)**, and **Community Support (CS)**.

For **SWB**, the Life Satisfaction subscale shows excellent reliability, with a Cronbach’s Alpha of 0.872, indicating strong internal consistency. The Positive Emotions and Negative Emotions subscales also demonstrate satisfactory reliability, with Cronbach’s alpha values of 0.821 and 0.858, respectively, highlighting the consistency of these measures.

The **PA** scale reveals high internal consistency across its subscales. The **frequency** subscale has the highest Cronbach’s alpha (0.911), followed by **intensity** (0.810) and **time** (0.784). The **Type** subscale also demonstrates good reliability (α = 0.803), suggesting that all dimensions of physical activity are reliably measured.

For **ASE**, the subscales assessing Health Maintenance, Psychological Adaptation, and Social Participation exhibit strong internal consistency, with Cronbach’s alpha values ranging from 0.868 to 0.905, indicating the reliability of the aging self-efficacy measures across these domains.

Finally, the **CS** subscales, which include emotional support, tool support, information support, and participation support, all show excellent reliability, with Cronbach’s alpha values ranging from 0.849 to 0.902. This indicates that the items across these subscales are highly consistent in measuring different types of community support.

In conclusion, the measurement scales in this study demonstrate strong internal consistency, with Cronbach’s alpha values consistently above 0.80, suggesting that the scales are reliable and valid for assessing the constructs of **SWB**, **PA**, **ASE**, and **CS**.
