# Supplementary material for: Parallel and chain mediating effects of physical activity and aging self-efficacy: community support and subjective well-being among Pre-K–9 teachers in Dongguan
Source: BMC Public Health. 2026 Jul 28;26:3312. doi: 10.1186/s12889-026-28694-2 (PMC13753292; doi:10.1186/s12889-026-28694-2)
Supplement: Supplementary file 4 [file 12889_2026_28694_MOESM3_ESM.docx]

**Appendix Table 3.** KMO and Bartlett's Test^a^

| Kaiser-Meyer-Olkin Measure of Sampling Adequacy. | | 0.899 |
| --- | --- | --- |
| Bartlett's Test of Sphericity | Approx. Chi-Square | 5639.156 |
|  | df | 91 |
|  | Sig. | <0.001 |

**Appendix Table 3** presents the results of the **Kaiser-Meyer-Olkin (KMO) measure of sampling adequacy** and **Bartlett's test of sphericity,** both of which are crucial for evaluating the suitability of the data for factor analysis.

The **KMO Measure of Sampling Adequacy** is reported as 0.899, which is considered excellent according to commonly accepted thresholds. A KMO value above 0.80 suggests that the correlations between variables are sufficiently large for factor analysis, indicating that the data is suitable for such a procedure.

**Bartlett's Test of Sphericity** tests the null hypothesis that the correlation matrix is an identity matrix, meaning that the variables are unrelated. The result shows an approximate chi-square value of 5639.156 with 91 degrees of freedom (df) and a p-value of less than 0.001. This significant result (p < 0.001) indicates that the variables are indeed correlated and that factor analysis is appropriate for the dataset.

In conclusion, the KMO value of 0.899 and the significant result of Bartlett's Test of Sphericity confirm that the data is suitable for factor analysis, supporting the validity of subsequent factor extraction procedures.
