# Supplementary material for: Parallel and chain mediating effects of physical activity and aging self-efficacy: community support and subjective well-being among Pre-K–9 teachers in Dongguan
Source: BMC Public Health. 2026 Jul 28;26:3312. doi: 10.1186/s12889-026-28694-2 (PMC13753292; doi:10.1186/s12889-026-28694-2)
Supplement: Supplementary file 5 [file 12889_2026_28694_MOESM4_ESM.docx]

**Appendix Table 4**. Total Variance Explained

| Component | Extraction Sums of Squared Loadings | | | Rotation Sums of Squared Loadings | | |
| --- | --- | --- | --- | --- | --- | --- |
|  | Total | % of Variance | Cumulative % | Total | % of Variance | Cumulative % |
| 1 | 15.515 | 20.966 | 20.966 | 7.080 | 9.568 | 9.568 |
| 2 | 5.084 | 6.871 | 27.837 | 2.801 | 3.785 | 13.353 |
| 3 | 2.907 | 3.928 | 31.765 | 2.612 | 3.530 | 16.883 |
| 4 | 2.758 | 3.727 | 35.492 | 2.605 | 3.520 | 20.403 |
| 5 | 2.352 | 3.179 | 38.671 | 2.600 | 3.514 | 23.917 |
| 6 | 2.280 | 3.081 | 41.752 | 2.542 | 3.435 | 27.352 |
| 7 | 2.206 | 2.981 | 44.733 | 2.513 | 3.396 | 30.748 |
| 8 | 2.034 | 2.749 | 47.482 | 2.490 | 3.364 | 34.112 |
| 9 | 1.891 | 2.555 | 50.038 | 2.467 | 3.333 | 37.446 |
| 10 | 1.631 | 2.205 | 52.242 | 2.435 | 3.290 | 40.736 |
| 11 | 1.608 | 2.173 | 54.415 | 2.411 | 3.259 | 43.995 |
| 12 | 1.398 | 1.889 | 56.304 | 2.387 | 3.226 | 47.220 |
| 13 | 1.368 | 1.849 | 58.153 | 2.372 | 3.206 | 50.426 |
| 14 | 1.341 | 1.812 | 59.965 | 2.263 | 3.059 | 53.484 |
| 15 | 1.289 | 1.742 | 61.707 | 2.254 | 3.046 | 56.531 |
| 16 | 1.216 | 1.644 | 63.351 | 2.223 | 3.004 | 59.534 |
| 17 | 1.157 | 1.564 | 64.915 | 2.139 | 2.890 | 62.424 |
| 18 | 1.065 | 1.439 | 66.354 | 2.000 | 2.703 | 65.127 |
| 19 | 1.060 | 1.432 | 67.786 | 1.867 | 2.523 | 67.650 |
| 20 | 1.012 | 1.367 | 69.153 | 1.113 | 1.503 | 69.153 |
| Extraction Method: Principal Component Analysis. | | | | | | |

**Appendix Table 4** reports the results of **Principal Component Analysis (PCA)**, detailing the **total variance explained** by the extracted and rotated components. Using the **Kaiser criterion**, the first 20 components were retained for further analysis.

The first component explains **20.97%** of the total variance, highlighting the presence of a dominant latent dimension. The cumulative variance explained by the first five components reaches **38.67%**, and by the tenth component, this increases to **52.24%**. After rotation, the first component accounts for **9.57%** of the variance, and the cumulative variance explained by the first ten components rises to **40.74%**. The **cumulative variance** explained by all twenty components remains at **69.15%**, both before and after rotation, suggesting a well-structured factor model.

These findings indicate that a small number of components explain a substantial portion of the variance, confirming the adequacy of the factor structure. The rotated components provide a more interpretable distribution of variance without altering the total explained variance. This supports the reliability of the factor solution and its suitability for further analysis.
