# Supplementary material for: Parallel and chain mediating effects of physical activity and aging self-efficacy: community support and subjective well-being among Pre-K–9 teachers in Dongguan
Source: BMC Public Health. 2026 Jul 28;26:3312. doi: 10.1186/s12889-026-28694-2 (PMC13753292; doi:10.1186/s12889-026-28694-2)
Supplement: Supplementary file 6 [file 12889_2026_28694_MOESM5_ESM.docx]

# Questionnaire

Dear Teacher,

Hello! This questionnaire is for academic research purposes. It is anonymous, and the data will be used solely for statistical analysis and will be kept strictly confidential. There are no right or wrong answers. Please respond truthfully based on your actual circumstances. Thank you for your support and cooperation!

**Part I: Demographic Characteristics**

1. Gender

A. Male

B. Female

2. Age

A. 25 years old and under

B. 26–35 years old

C. 36–45 years old

D. 46–55 years old

E. 56 years old and over

3. Marital Status

A. Unmarried

B. Married

C. Divorced

D. Widowed

4. Educational Background

A. Associate Degree or Below

B. Bachelor's Degree

C. Master's Degree and Above

5. Years of Teaching Experience

A. 5 Years or Less

B. 6–10 Years

C. 11–15 Years

D. 16–20 Years

E. 21 Years or More

6. Type of School Taught At

A. Public kindergartens

B. Private kindergartens

C. Public elementary schools

D. Private elementary schools

E. Public middle schools

F. Private middle schools

7. Position Type

A. Full-time Teacher

B. Administrative and teaching assistants

C. Support staff and healthcare

8. Average Monthly Income

A. ¥3,000 or below

B. ¥3,001–5,000

C. ¥5,001–8,000

D. ¥8,001–12,000

E. ¥12,001 and above

9. Do you hold positions such as homeroom teacher/grade level coordinator?

A. Yes

B. No

**Part II: Community Support**

Instructions: Please select based on your genuine feelings over the past month.

Scoring: A. Strongly disagree (1 point); B. Somewhat disagree (2 points); C. Neutral (3 points); D. Somewhat agree (4 points); E. Strongly agree (5 points)

(1) Emotional Support

1. I feel the community residents treat me with friendliness and respect.

A. Strongly disagree

B. Disagree

C. Neutral

D. Agree

E. Strongly agree

2. The community provides me with care and emotional understanding.

A. Strongly disagree

B. Disagree

C. Neutral

D. Agree

E. Strongly agree

3. When facing troubles, there are people in the community willing to listen.

A. Strongly disagree

B. Disagree

C. Neutral

D. Agree

E. Strongly agree

(2) Instrumental Support

4. The community provides practical services such as fitness and health programs.

A. Strongly disagree

B. Disagree

C. Neutral

D. Agree

E. Strongly agree

5. Community public sports and recreational facilities are convenient to use.

A. Not at all true

B. Somewhat untrue

C. Neutral

D. Somewhat true

E. Very true

6. When I encounter difficulties, the community can provide practical assistance.

A. Not at all true

B. Somewhat untrue

C. Neutral

D. Somewhat true

E. Very true

(3) Information Support

7. The community promptly disseminates information related to health, exercise, and elderly care.

A. Very Unsatisfactory

B. Somewhat Unsatisfactory

C. Average

D. Satisfactory

E. Very Satisfactory

8. I can obtain scientifically sound health and wellness knowledge from the community.

A. Very Unsatisfactory

B. Somewhat Unsatisfactory

C. Average

D. Satisfactory

E. Very Satisfactory

9. The community promotes active aging and healthy living.

A. Strongly disagree

B. Disagree

C. Neutral

D. Agree

E. Strongly agree

(4) Participation Support

10. The community frequently organizes cultural, recreational, and health-related activities.

A. Strongly disagree

B. Disagree

C. Neutral

D. Agree

E. Strongly agree

11. I have opportunities to participate in various activities organized by the community.

A. Strongly disagree

B. Disagree

C. Neutral

D. Agree

E. Strongly agree

12. The community provides platforms for residents to participate in public activities.

A. Strongly disagree

B. Disagree

C. Neutral

D. Agree

E. Strongly agree

**Part III: Physical Activity**

Instructions: Please select based on your actual participation in physical activities over the past month.

Scoring: A. Not at all applicable (1 point); B. Not very applicable (2 points); C. Somewhat applicable (3 points); D. Fairly applicable (4 points); E. Very applicable (5 points);

(1) Activity Intensity

1. My physical activities cause noticeable increases in heart rate and rapid breathing.

A. Strongly disagree

B. Somewhat disagree

C. Neutral

D. Somewhat agree

E. Strongly agree

2. I engage in activities that induce sweating and moderate fatigue.

A. Strongly disagree

B. Somewhat disagree

C. Neutral

D. Somewhat agree

E. Strongly agree

3. My physical activities reach moderate or higher exercise intensity.

A. Strongly disagree

B. Disagree

C. Neutral

D. Agree

E. Strongly agree

(2) Activity Frequency

4. I engage in regular physical exercise every week.

A. Strongly disagree

B. Disagree

C. Neutral

D. Agree

E. Strongly agree

5. The number of times I participate in physical activities each week is relatively stable.

A. Not at all true

B. Somewhat untrue

C. Neutral

D. Somewhat true

E. Very true

6. I maintain physical exercise 3 or more times per week.

A. Not at all true

B. Somewhat untrue

C. Neutral

D. Somewhat true

E. Very true

(3) Activity Time

7. Each of my physical activity sessions lasts 30 minutes or longer.

A. Strongly disagree

B. Somewhat disagree

C. Neutral

D. Somewhat agree

E. Strongly agree

8. I can complete a continuous period of physical exercise without easily interrupting it.

A. Strongly disagree

B. Somewhat disagree

C. Neutral

D. Somewhat agree

E. Strongly agree

9. The duration of each exercise session meets my physical activity needs.

A. Strongly disagree

B. Disagree

C. Neutral

D. Agree

E. Strongly agree

(4) Activity Type

10. I participate in a variety of sports such as running, ball games, fitness training, and yoga.

A. Strongly disagree

B. Disagree

C. Neutral

D. Agree

E. Strongly agree

11. I choose exercise types suitable for my physical condition.

A. Strongly disagree

B. Disagree

C. Neutral

D. Agree

E. Strongly agree

12. I flexibly choose different exercise methods based on my interests.

A. Strongly disagree

B. Disagree

C. Neutral

D. Agree

E. Strongly agree

**Part IV: Aging Self-Efficacy**

Instructions: Please assess your level of confidence in coping with aging, maintaining health, and preserving your ability to live independently in the future.

Scoring: A. Not at all confident (1 point); B. Not very confident (2 points); C. Somewhat confident (3 points); D. Fairly confident (4 points); E. Very confident (5 points);

(1) Health Maintenance Self-Efficacy

1. I am confident in maintaining a healthy lifestyle long-term.

A. Not at all confident

B. Not very confident

C. Somewhat confident

D. Confident

E. Very confident

2. I am confident in preventing and managing the onset and progression of chronic diseases.

A. Not at all confident

B. Not very confident

C. Somewhat confident

D. Confident

E. Very confident

3. I am confident in maintaining good physical function and mobility.

A. Not at all confident

B. Not very confident

C. Somewhat confident

D. Quite confident

E. Very confident

(2) Psychological Adaptation Self-Efficacy

4. I am confident in maintaining a positive and optimistic mindset toward aging.

A. Not at all confident

B. Not very confident

C. Somewhat confident

D. Confident

E. Very confident

5. I am confident in self-regulating my emotions and alleviating stress and anxiety.

A. Not at all confident

B. Not very confident

C. Somewhat confident

D. Confident

E. Very confident

6. I am confident in calmly handling changes that may occur during old age.

A. Not at all confident

B. Not very confident

C. Somewhat confident

D. Quite confident

E. Very confident

(3) Social Participation Self-Efficacy

7. I am confident in maintaining stable, positive interpersonal relationships.

A. Not at all confident

B. Not very confident

C. Somewhat confident

D. Quite confident

E. Very confident

8. I am confident in continuing to participate in social or group activities.

A. Not at all confident

B. Not very confident

C. Somewhat confident

D. Quite confident

E. Very confident

9. I am confident in maintaining independent living and a sense of self-worth in later life.

A. Not at all confident

B. Not very confident

C. Somewhat confident

D. Quite confident

E. Very confident

**Part V: Subjective Well-Being**

Instructions: Please select based on your overall feelings over the past month.

Scoring: A. Strongly disagree (1 point); B. Disagree (2 points); C. Neutral (3 points); D. Agree (4 points); E. Strongly agree (5 points);

(1) Life Satisfaction

1. I am satisfied with my overall life situation at present.

A. Strongly disagree

B. Somewhat disagree

C. Neutral

D. Somewhat agree

E. Strongly agree

2. My life is close to my ideal state.

A. Strongly disagree

B. Somewhat disagree

C. Neutral

D. Somewhat agree

E. Strongly agree

3. I feel fulfilled with my work and life situation.

A. Strongly disagree

B. Somewhat disagree

C. Neutral

D. Somewhat agree

E. Strongly agree

(2) Positive Emotions

4. Most of the time, I feel happy and fulfilled.

A. Strongly disagree

B. Disagree

C. Neutral

D. Agree

E. Strongly agree

5. I frequently experience joy and happiness in life.

A. Strongly disagree

B. Disagree

C. Neutral

D. Agree

E. Strongly agree

6. I feel hopeful about my future work and life.

A. Strongly disagree

B. Disagree

C. Neutral

D. Agree

E. Strongly agree

(3) Negative Emotions

7. I often feel anxious or irritable.

A. Strongly disagree

B. Disagree

C. Neutral

D. Agree

E. Strongly agree

8. I easily feel down or gloomy.

A. Strongly disagree

B. Disagree

C. Neutral

D. Agree

E. Strongly agree

9. I often feel exhausted due to high stress levels.

A. Strongly disagree

B. Disagree

C. Neutral

D. Agree

E. Strongly agree
